# Idiopathic Internal Jugular Vein and Subclavian Vein Thrombosis: A Rare Case Report

**DOI:** 10.7759/cureus.4005

**Published:** 2019-02-04

**Authors:** Ankit Agrawal, Divyansh Bajaj, Megan Ruben, John George

**Affiliations:** 1 Internal Medicine, Rutgers Robert Wood Johnson Medical School / Saint Peter's University Hospital, New Brunswick, USA; 2 Internal Medicine, Saint Vincent Medical Center, Bridgeport, USA; 3 Internal Medicine, Bridgeport Hospital, Milford, USA

**Keywords:** venous thrombosis, anticoagulation, upper extremity

## Abstract

Venous thrombosis is a vascular disorder which is a consequence of Virchow’s triad: hypercoagulability, venous stasis, and endothelial injury. While lower extremity deep venous thrombosis is common, upper torso thrombosis is a rare clinical condition and usually a complication of central venous catheterization or malignancy-related paraneoplastic syndromes. Herein, we present a rare case of a 64-year-old male who presented with right upper extremity and right facial swelling who was found to have a thrombus in the right internal jugular vein and right subclavian vein with no predisposing factors. He was successfully treated with anticoagulation without any complications.

## Introduction

Venous thrombosis is a vascular disorder which is a consequence of Virchow’s triad: hypercoagulability, venous stasis, and endothelial injury. While lower extremity deep venous thrombosis is common, upper torso thrombosis is a rare clinical condition and is usually a complication of central venous catheterization or malignancy-related paraneoplastic syndromes. The incidence is reported to be 5% - 10% of all cases of deep venous thrombosis (DVT) [1]. Here, we present a rare case of idiopathic internal jugular and subclavian vein thrombosis successfully treated with anticoagulation without any complication.

## Case presentation

**Figure 1:**
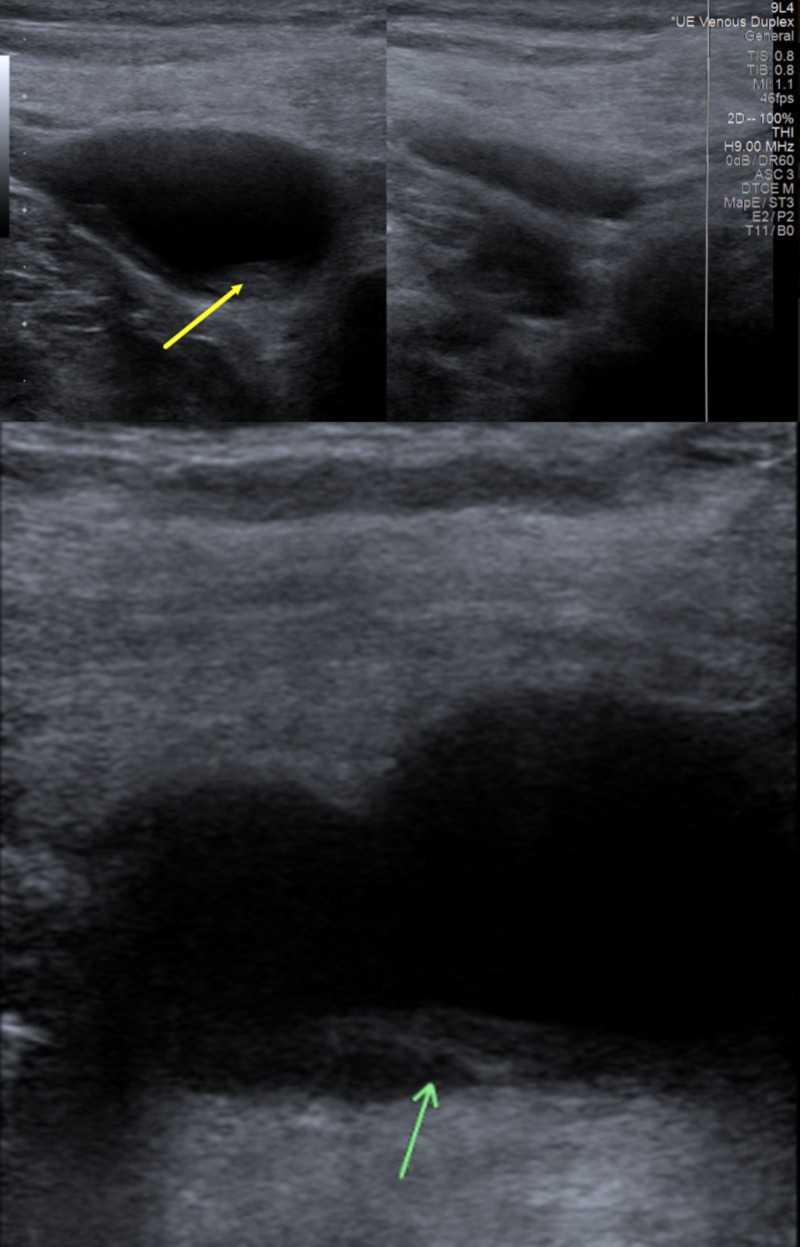
Venous duplex ultrasonography of the neck veins The venous duplex ultrasonographic study showed a non-occlusive thrombus (yellow arrow) in proximal right internal jugular vein in the top panel with a clear view of the thrombus (green arrow) in a magnified view in the bottom panel.

**Figure 2:**
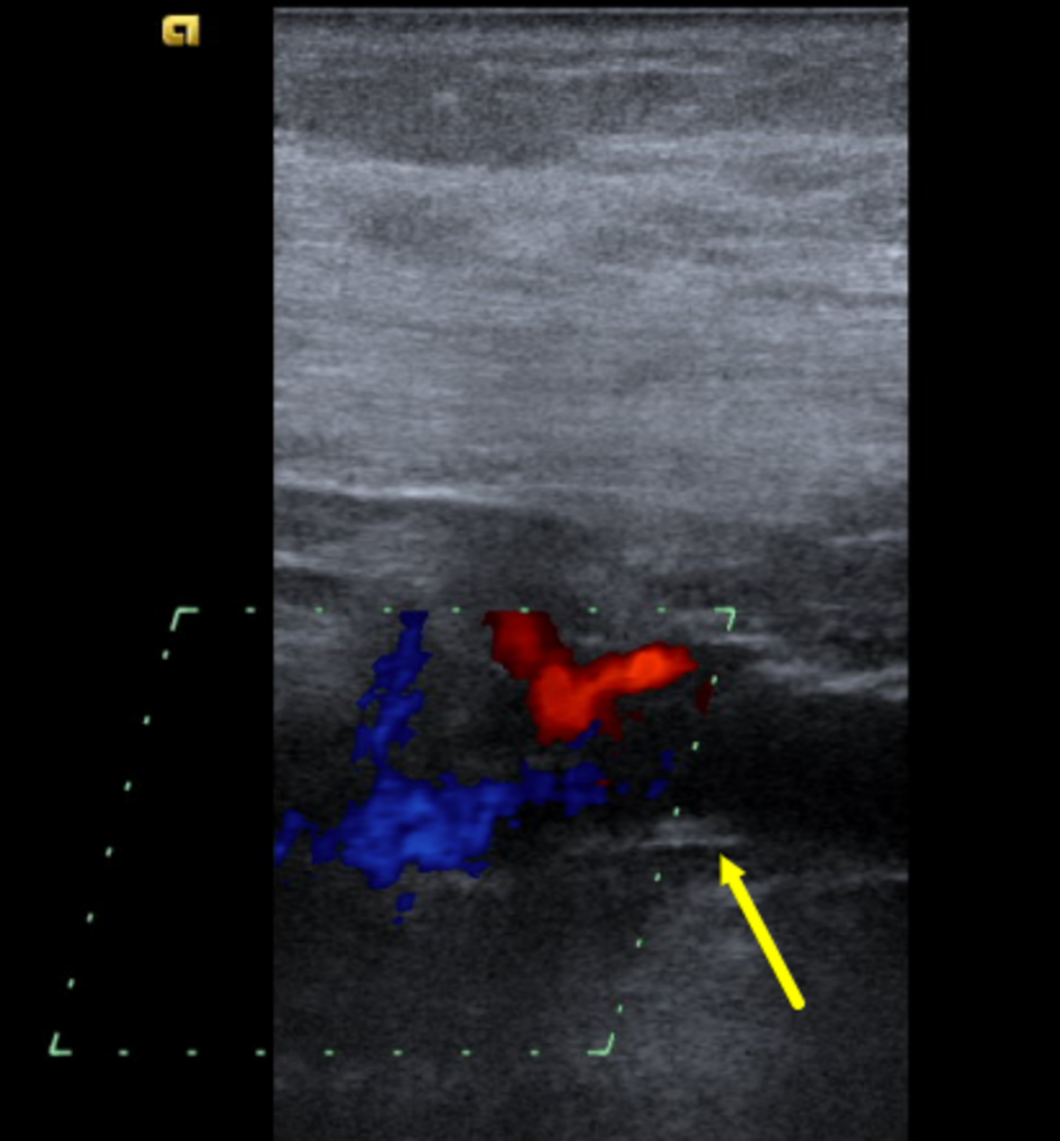
Venous duplex ultrasonography of the neck veins in color mode The venous duplex ultrasonography in color mode showed a non-occlusive thrombus in right subclavian vein (yellow arrow) at the confluence of cephalic vein. A mildly diminished flow is noted in the study.

A 64-year-old Caucasian male with a medical history of hypertension and symptomatic bradycardia status-post pacemaker implantation presented to the emergency department with the chief complaint of swelling of the right upper extremity for three weeks. It progressed to a generalized swelling of the right shoulder and right side of the neck, restricting his arm movements. He also voiced the concern of worsening shortness of breath which started with the swelling. He did not report any personal or family history of thrombophilia. There was no previous history of any excessive upper extremity exertion or catheterization in the neck. Vital signs on presentation were a blood pressure of 127/61 mmHg, pulse rate of 79/minute, good volume, regular rhythm with no radio-radial or radio-femoral delay, respiratory rate of 19/min with a saturation of 93% on 3-liters nasal cannula, and temperature of 97.5^o^F. Physical examination revealed right upper extremity swelling without any sensory or motor deficits, right-sided neck swelling, and right-sided facial plethora. Examination of the contralateral arm, as well as the cardiovascular and respiratory systems, was normal. Biochemical investigations were within normal limits. The patient underwent a right upper extremity duplex ultrasonography which revealed an acute non-occlusive thrombus in the proximal right internal jugular vein (Figure 1) and right subclavian vein (Figure 2) at the cephalic vein confluence. At this point, Factor V Leiden, anti-thrombin III, protein C, and protein S levels were ordered which were normal. The patient was admitted to the medical ward and an intravenous heparin infusion was initiated. Chest radiography did not reveal any cervical rib, and computed tomography (CT) pulmonary angiography showed no evidence of pulmonary embolism. The swelling improved over the course of two days, and the patient was switched to oral apixaban for anticoagulation. He was discharged on apixaban for six months, and a complete resolution of his signs and symptoms was noted at his three-month follow-up examination.

## Discussion

The upper extremity venous anatomy is divided into the superficial system and deep venous system. The latter includes the paired ulnar, radial, and interosseous veins in the forearm, paired brachial veins in the upper arm, and the axillary vein, which, in turn, becomes the subclavian vein at the teres major muscle. Venous thrombosis is an intravascular condition which happens as a result of alteration of blood constituents (hypercoagulable states), alterations in the blood flow, and vascular endothelial injuries. Superficial venous system thrombosis is less severe than the deep vein thrombosis (DVT). Upper extremity deep vein thrombosis (UEDVT) is a rare phenomenon carrying a high morbidity and mortality rate of 29% - 40% [2] due to complications, such as pulmonary embolism, the incidence of which is 12% from upper extremity thrombus [3] and intracranial extension of the thrombus. Primary UEDVT, which is less common than secondary UEDVT, constitutes 20% of all UEDVT [2]. Risk factors for primary thrombosis include strenuous upper extremity activities or thoracic outlet anatomic abnormalities. Malignancy, indwelling catheters, pacemaker implantation, defibrillators, hypercoagulable states like thrombophilia, antiphospholipid syndrome [4], intravenous drug abuse, trauma, and infections constitute the secondary risk factors. Clinical signs include edema or cyanosis of the affected arm and neck. Low-grade fever can also be present, mimicking a soft tissue neck infection. Neurological signs, such as paresthesias, denote brachial plexus compression at the thoracic outlet. The arterial system is usually intact and requires emergent treatment if compromised as a result of venous congestion. Our patient did not have any signs or symptoms of limb ischemia. He had a pacemaker placed on the left side, which was in contrast to the symptoms that were confined to the right arm and neck only.

Duplex ultrasonography is the diagnostic modality of choice as it is noninvasive and inexpensive. It has a sensitivity ranging from 78% to 100% and a specificity of 82% to 100% [5]. Catheter-based digital subtraction venography predicts excellent venous anatomy but is not used widely because of its invasive nature. It comes into the role when noninvasive diagnostic choices demonstrate equivocal findings. Other modalities include computed tomographic or magnetic resonance venography which can be used in equivocal cases. Duplex ultrasound demonstrated the evidence of thrombus in our case, and hence no further investigations were done for further clarification. 

The goal of treatment should be the resolution of the symptoms and prevention of recurrent episodes and further complications. Three different management pathways can be chosen - anticoagulation, thrombolysis, or thoracic outlet surgical decompression. The anticoagulation choice can be either unfractionated heparin, low-molecular-weight heparin, or oral anticoagulants, such as apixaban, dabigatran, or rivaroxaban. Use of edoxaban has also been established in the literature [2]. Catheter-directed thrombolytic therapy causes rapid dissolution of the thrombus and restores venous function, reducing the edema and endothelial inflammation. It is successful in 62% to 84% of the cases [6] and the “fresher” the clot, the higher is the success rate [7]. Thoracic outlet decompression surgery is done in select patients with chronic thrombosis, patients with abnormal thoracic outlet anatomy, or recurrent thrombosis despite thrombolysis. It reduces long-term morbidity. Surgical options include first rib resection, cervical rib resection, partial anterior scalenectomy, or resection of the costoclavicular ligament. Our patient was successfully treated with anticoagulation with no recurrence on follow-up.

## Conclusions

In conclusion, spontaneous internal jugular vein and subclavian vein thrombosis is an extremely rare condition. Nevertheless, secondary etiologies must always be ruled out during the workup. There is no established management algorithm in such clinical scenarios and treatment is usually experience-based. Our case highlights the increasing prevalence of this rare entity and advises clinicians to individualize the management plan based on the severity and extent of the thrombosis.

## References

[1] Muñoz FJ, Mismetti P, Poggio R, Valle R, Barrón M, Guil M, Monreal M (2008). Clinical outcome of patients with upper-extremity deep vein thrombosis: results from the RIETE Registry. Chest.

[2] Toratani M, Hayashi A, Nishiyama N (2017). Thrombosis in an internal jugular vein and an upper limb deep vein treated with edoxaban. Intern Med.

[3] Nemmers DW, Thorpe PE, Knibbe MA, Beard DW (1990). Upper extremity venous thrombosis. Case report and literature review. Orthop Rev.

[4] Al-Zoubi NA (2018). Spontaneous internal jugular vein thrombosis as primary presentation of antiphospholipid syndrome: case report. Vasc Health Risk Manag.

[5] Chin EE, Zimmerman PT, Grant EG (2005). Sonographic evaluation of upper extremity deep venous thrombosis. J Ultrasound Med.

[6] Illig KA, Doyle AJ (2010). A comprehensive review of Paget-Schroetter syndrome. J Vasc Surg.

[7] Vik A, Holme PA, Singh K, Dorenberg E, Nordhus KC, Kumar S, Hansen JB (2009). Catheter-directed thrombolysis for treatment of deep venous thrombosis in the upper extremities. Cardiovasc Intervent Radiol.

